# Efficacy of Combined Laparoscopic and Hysteroscopic Repair of Post-Cesarean Section Uterine Diverticulum: A Retrospective Analysis

**DOI:** 10.1155/2016/1765624

**Published:** 2016-03-15

**Authors:** Cuilan Li, Shiyan Tang, Xingcheng Gao, Wanping Lin, Dong Han, Jinguo Zhai, Xuetang Mo, Lee Jaden Gil Yu Kang Zhou

**Affiliations:** ^1^The Third Affiliated Hospital, Guangzhou Medical University, Key Laboratory for Major Obstetric Diseases of Guangdong Province, Key Laboratory of Reproduction and Genetics of Guangdong Higher Education Institutes, Guangzhou, Guangdong 510145, China; ^2^St. Louis School, 179 Third Street, Sai Ying Pun, Hong Kong

## Abstract

*Background*. Diverticulum, one of the long-term sequelae of cesarean section, can cause abnormal uterine bleeding and increase the risk of uterine scar rupture. In this study, we aimed to evaluate the efficacy of combined laparoscopic and hysteroscopic repair, a newly occurring method, treating post-cesarean section uterine scar diverticulum.* Methods*. Data relating to 40 patients with post-cesarean section uterine diverticulum who underwent combined laparoscopic and hysteroscopic repair were retrospectively analyzed. Preoperative clinical manifestations, size of uterine defects, thickness of the lower uterine segment (LUS), and duration of menstruation were compared with follow-up findings at 1, 3, and 6 months after surgery.* Results*. The average preoperative length and width of uterine diverticula and thickness of the lower uterine segment were recorded and analyzed. The average durations of menstruations at 1, 3, and 6 months after surgery were significantly shorter than the preoperative one (*p* < 0.05), respectively. At 6 months after surgery, the overall success improvement rate of surgery was 90% (36/40). Three patients (3/40 = 7.5%) developed partial improvement, and 1/40 (2.5%) was lost to follow-up.* Conclusions*. Our findings showed that combined treatment with laparoscopy and hysteroscopy was an effective method for the repair of post-cesarean section uterine diverticulum.

## 1. Introduction

Over the last few decades, rates of delivery by cesarean section have continued to increase, which has attracted much attention [1, 2]. Cesarean section carries long-term sequelae, which can adversely affect subsequent pregnancies. In a recent study, 64.5% of patients who underwent cesarean section developed uterine incision diverticulum within 6 to 12 weeks after surgery, which can cause abnormal uterine bleeding [3]. In addition, pregnancy in the diverticulum increases the risk of uterine scar rupture, endangering the life of both the mother and fetus [4].

Guidelines for the diagnosis and treatment of uterine diverticulum are unclear. Currently, the two main treatment options include conservative treatment based on combined estrogen and progesterone therapy and surgical repair of the diverticulum. Oral contraceptives, which are suitable for those unable to tolerate surgery, can reduce bleeding associated with the diverticulum by controlling endometrial proliferation. However, although oral contraceptives can improve microcirculation, withdrawal of the treatment may cause recurrence of irregular bleeding. In addition, studies have indicated that, for most patients undergoing conservative hormone therapy, symptoms are alleviated only temporarily and the risk of diverticulum pregnancy continues to persist. Eventually, most patients with uterine diverticulum require surgical repair [5].

Recently, several investigators have proposed a solution to development of uterine diverticulum. Api et al. showed that hysteroscopic treatment may correct the scar defect, although this technique does not strengthen the uterine wall and does not appear to decrease the potential risk of dehiscence or rupture in subsequent pregnancies [6]. Another report described that cavities were identified in the uterine horns and cervical diverticulum by hysteroscopy and the diagnosis was confirmed by laparoscopy after treatment [7]. Moreover, in a study by Li et al., patients who underwent either laparoscopic or hysteroscopic surgical repair showed improvement in their symptoms following surgical treatment, depending on whether the residual myometrial thickness was less than or ≥3.5 mm and the defect accounted for less than or ≥50% of the anterior uterine wall [8]. In addition, Nirgianakis et al. recently reported the feasibility, safety, and effectiveness of the rendezvous technique, a minimally invasive surgical approach combining laparoscopy and hysteroscopy, in women with cesarean section scar defects [9].

In this study, we aimed to examine the feasibility of treating post-cesarean section scar diverticulum using a combination of laparoscopic and hysteroscopic uterine repair.

## 2. Methods

### 2.1. Study Location

The study was conducted at the Guangzhou Institute of Obstetrics and Gynecology, The Third Affiliated Hospital, Guangzhou Medical University, Guangzhou, China. The study protocol was approved by the Ethical Committee at Guangzhou Medical University.

### 2.2. Study Subjects

We conducted a retrospective analysis of data relating to 40 patients with post-cesarean section uterine diverticulum who underwent combined hysteroscopic and laparoscopic repair between January 2012 and June 2015. The patients were examined at the end of menstruation (or on the tenth day for patients with menstruation lasting >10 days) by transvaginal three-dimensional (3D) color Doppler ultrasound. The diagnosis of post-cesarean section uterine diverticulum was made on the basis of the following: (1) uneven distribution of the uterine wall thickness and absence of smooth area over the lower section of the myometrium at the site of the cesarean section scar; (2) detection of anechoic space (with or without fluid), at least 2 mm deep, at the site of the cesarean section scar, plus myometrial thinning of the anterior uterine wall; and (3) vascular hyperplasia and blood clots in the uterine serosa, with an arched or dome-shaped cesarean incision site [6].

Patients with diverticulum associated with abdominal incisional hernia or congenital abdominal wall dysplasia were excluded from the study.

### 2.3. Laparoscopic and Hysteroscopic Repair

All patients underwent combined laparoscopic and hysteroscopic repair within 1 week after menstruation. All procedures were conducted under general anesthesia, and patients were placed in the Trendelenburg (head-down) position.

**Figure 1:**
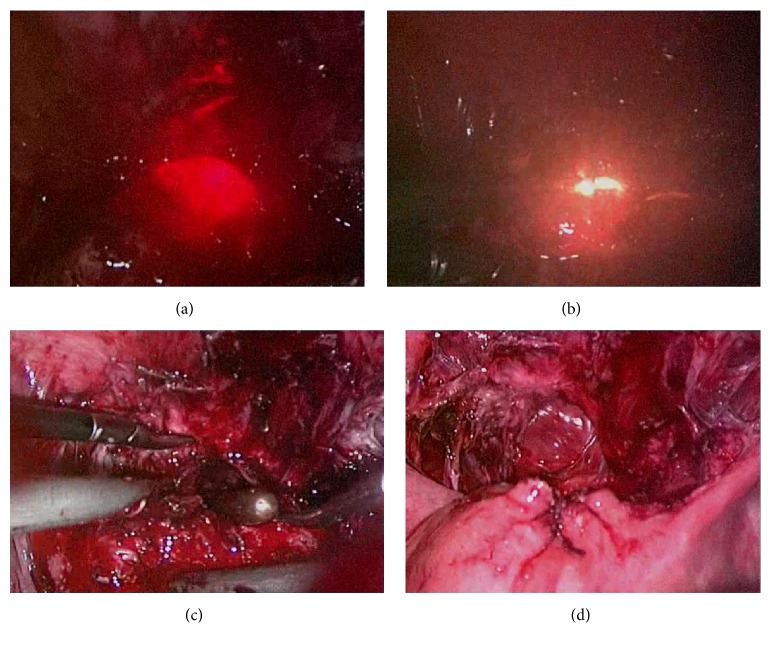
Post-cesarean section uterine diverticulum treated using a combination of laparoscopy and hysteroscopy. (a) Laparoscopic view of the weakest part of the post-cesarean section diverticulum using an external orange-red light source. (b) Use of an electric coagulation hook to open the diverticulum under hysteroscopic guidance. (c) Cutting and trimming the full length of the uterine diverticulum. (d) 2-0 absorbable stitch used for full-thickness suturing and closure of the peritoneum over the bladder.

An ultrasonic scalpel was used to incise the peritoneal fold over the bladder, and the bladder was pushed down to 2 cm below the lower edge of the diverticulum with the help of duckbill pliers. Under hysteroscopic examination, the operating surgeon identified the presence of diverticular mucosal hyperplasia, which appeared partially white, to confirm the location and size of the post-cesarean section uterine diverticulum. In addition, a surgical assistant used an external orange-red light source to locate the weakest part of the diverticulum. The surgeon then used an electric coagulation hook to open the diverticulum laparoscopically. The uterine diverticulum was cut in full length, the wound trimmed, and 2-0 absorbable stitch used to perform full-thickness suture. The peritoneum covering the bladder was closed after the repair (Figure 1).

### 2.4. Evaluation and Follow-Up

All 40 patients were followed up postoperatively at 1, 3, and 6 months after surgery.

Surgical outcomes were categorized as follows: (I) improvement: postoperative menstrual cycle shortened to <7 days or to 2 days shorter than the previous cycle before surgery, as well as alleviation of irregular bleeding, abdominal pain, and vaginal discomfort, disappearance or reduction in size of the liquid dark area of the uterine scar plus improved thickness of the serosal layer, including at the thinnest part of the uterine scar diverticulum, on transvaginal 3D Doppler ultrasound; (II) partial improvement: shortening of the menstrual period by ≥2 days with improvement in irregular bleeding or reappearance of the liquid dark area of the uterine scar with abnormal serosal layer thickness on 3D Doppler ultrasound; and (III) no improvement: no improvement in postmenstrual spotting phenomenon or presence of abdominal pain and vaginal secretions or no narrowing of the liquid dark area of the anterior uterine wall on transvaginal 3D Doppler ultrasound.

### 2.5. Statistical Analysis

Student's* t*-test was used to assess intergroup differences with respect to size of the uterine scar defect, thickness of the lower uterine segment (LUS), and duration of the menstrual period, before and after surgical treatment. Statistical analysis was performed using SPSS software version 18.0 (IBM, Chicago, IL, USA) and Systat SigmaPlot version 12.5 (Systat Software Inc., San Jose, CA, USA).

## 3. Results

The average age of study subjects was 30.7 ± 6.6 years. Of the 40 patients, 26 (65%) had undergone a single previous delivery and 14 patients (35%) ≥2 deliveries, by cesarean section. A total of 19 patients (47.5%) experienced chronic menstrual pain in the absence of any obvious cause, and 12 patients (30%) had recurrent vaginitis. Uterine incision diverticulum was diagnosed in 8 patients (20%) upon ultrasound examination for secondary infertility.

Before surgery, 7 patients (17.5%) had undergone lysis of pelvic adhesions, and 6 (15%) had undergone endometrial polyp resection or treatment for sequelae of pelvic inflammatory disease. A total of 15 patients (37.5%) had undergone myomectomy, which was not associated with complications such as massive hemorrhage or uterine perforation. Doppler ultrasound data showed that the mean preoperative length, width, and thickness of the LUS were 14.4 ± 4.4 mm, 9.4 ± 3.0 mm, and 3.4 ± 1.2 mm, respectively.

**Figure 2:**
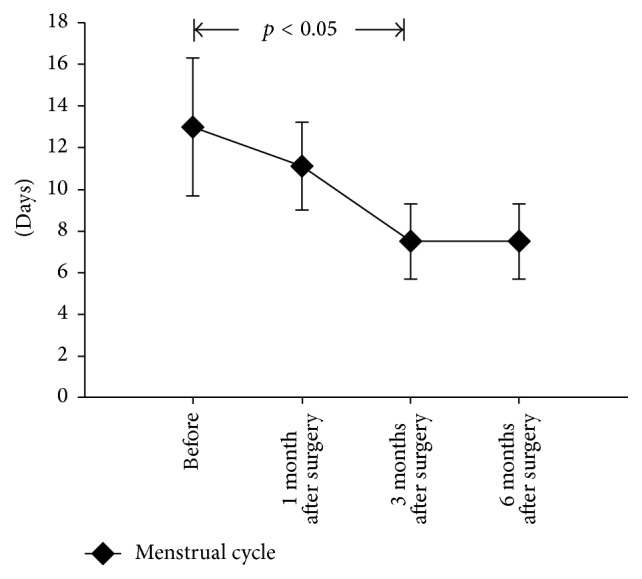
Preoperative and postoperative duration of menstruation at 1, 3, and 6 months after surgery.

According to surgery records, the average operating time and average intraoperative volume of bleeding were 122.4 ± 35.4 min (range 30–180 min) and 77.5 ± 24.7 mL (range 10–200 mL), respectively. The mean duration of menstruation before laparoscopy and hysteroscopy Lsc-Hsc was 13.0 ± 3.3 days (range 8–21 days). At 1, 3, and 6 months after surgery, the mean duration of the menstrual period decreased to 10.5 ± 2.1 days (range 6–18 days), 7.5 ± 1.8 days (range 5–12 days), and 7.6 ± 1.6 days (range 5–13 days), respectively, which was statistically significant, compared to the preoperative duration (*p* < 0.05) (Table 1, Figure 2). In addition, 37 patients (92.5%) showed improvement in the menstrual cycle postoperatively.

At 6 months postoperatively, 36 patients (90%) had improvement (surgical outcome I), 3 patients (7.5%) had partial improvement (surgical outcome II), and 1 patient (2.5%) was lost to follow-up (Table 2). A significant improvement in the duration of menstruation, compared to that preoperatively, was observed at 3 months after surgery (Table 3, Figure 1). A total of 3 patients still had diverticulum at the post-cesarean section scar after surgery.

## 4. Discussion

The endometrium and myometrium are prone to protrusion as a result of weakness of the LUS, which is referred to as uterine diverticulum [10]. Congenital uterine diverticulum is usually associated with congenital renal dysplasia. Acquired diverticulum is mainly caused by poor wound healing after cesarean section, with an incidence of 4–9% [11]. Complications of cesarean section, including poor wound healing, dysplastic endometrial capillary dilatation, and inflammatory tissue infiltration [12], increase the risk of expansion of the uterine incision. Postmenstrual bleeding is possibly the only obvious clinical manifestation of uterine incision diverticulum, whereas some patients also develop chronic dysmenorrhea. More often, the clinical manifestations are not so obvious, and the diverticulum is diagnosed only on ultrasound examination [13]. However, childbirth or pregnancy in those with uterine scar diverticulum can increase the risk of uterine rupture, endangering the life of both the mother and fetus [13–15].

Methods of operative repair of uterine diverticulum include hysteroscopy, laparoscopy, vaginal repair, and the combined use of hysteroscopy and laparoscopy. The choice of the surgical approach is mainly based on the clinical features and surgical skills of the operating team [16, 17]. Once a scar defect is confirmed, microsurgical reconstruction of the uterine diverticulum can partly attenuate symptoms such as postmenstrual spotting, abdominal pain, and infertility [17].

However, the microsurgical construction including hysteroscopy or laparoscopy alone has its shortcomings such as poor visualization and consequently low accuracy of diverticular orientation. Klemm et al. [15] proposed that ineffective drug therapy could be replaced by surgical repair using hysteroscopy combined with laparoscopy and that this combined approach is more effective and safe, as it allows superior visualization of the diverticulum, which, in turn, helps to improve the accuracy of orientation. A more recent study [16] reported significant clinical improvement in 14 patients with uterine incision diverticulum who underwent combined hysteroscopic and laparoscopic repair.

In our study, all 40 patients, who were diagnosed with post-cesarean section uterine diverticulum by transvaginal 3D Doppler ultrasound examination, underwent combined hysteroscopic and laparoscopic repair. All patients who had serosal thickening (2–5 mm) in the scar diverticulum manifested obvious clinical symptoms such as prolonged menstruation and abdominal pain. Symptomatic improvement was observed in all patients at 1 month after surgery. At 3 and 6 months after surgery, the regularity of menstrual cycles was improved in the majority of patients. In addition, the improvement rate of combined laparoscopic and hysteroscopic repair from our study was slightly higher (95%) than that previously reported for transvaginal repair (85.9%) [2]. Moreover, in our study, no major complications, such as massive bleeding or uterine perforation, were encountered during surgery.

The combined approach of hysteroscopy with laparoscopy offers many advantages. First, the bladder can be pushed down during laparoscopy to fully expose the diverticulum, thus minimizing the risk of iatrogenic injury to the bladder. Second, the condition of the abdominopelvic cavity can be visualized at laparoscopy, and the presence of chronic pelvic inflammatory disease detected and treated [2]. Third, hysteroscopy makes use of an orange-red light source for guidance to accurately determine the location and extent of the diverticulum. Fourth, after resection of the diverticulum at laparoscopy, surgical repair can be confirmed by hysteroscopy, thus minimizing the risk of complications [16]. Clearly two techniques are better than one, but not always, for combined approach of hysteroscopy with laparoscopy might have a higher fee and need more surgery time, and the operating surgeon must be familiar with the sight of diverticulum under both laparoscopy and hysteroscopy.

## 5. Conclusions

Our findings indicate combined laparoscopy and hysteroscopy repair as an appropriate and effective method for the diagnosis and repair of post-cesarean section uterine diverticulum. This technique is especially attractive in light of its main advantages as a combined approach for indication of exact extent and localization of the scar diverticulum and immediate assessment of repair. Further studies on a larger scale will certainly be needed to confirm our findings.

## Figures and Tables

**Table 1:** Baseline patient characteristics (*n* = 40).

	Number of patients	%
Age (years)	30.7 ± 6.6 (range 24–42)	
History of 1 cesarean section delivery	26	65
History of >2 cesarean section deliveries	14	35
Postmenstrual spotting	37	92.5
Abdominal pain	19	47.5
Recurrent vaginitis	12	30
Infertility	8	20
Duration of menstrual cycle (days)	13.0 ± 3.3 (range 8–21)	
Mean length of uterine diverticulum (mm)	14.4 ± 4.4 (range 6–26)	
Mean width of uterine diverticulum (mm)	9.4 ± 3.0 (range 5–14)	
Mean LUS^*∗*^ of uterine diverticulum (mm)	3.4 ± 1.2 (range 2–5)	

^*∗*^LUS, lower uterine segment.

**Table 2:** Surgical outcomes at follow-up (*n* = 40).

Time after surgery (months)	(I) Improvement (%)	(II) Partial improvement (%)	(III) No improvement (%)	Loss to follow-up (%)
1	30 (75)	9 (22.5)	1 (2.5)	0 (0)
3	34 (85)	5 (12.5)	0 (0)	1 (2.5)
6	36 (90)	3 (7.5)	0 (0)	1 (2.5)

**Table 3:** Intraoperative parameters and treatment outcomes.

	Number of patients	%
Average operation time (min)	122.4 ± 15.4 (range 30–180)	
Average volume of bleeding (mL)	77.5 ± 24.7 (range 10–200)	
Pelvic adhesion treatment (*n*)	7	17.5
Endometrial polyp resection or treatment for pelvic inflammatory disease sequelae (*n*)	6	15
Myomectomy (*n*)	15	37.5
Duration of menstruation at 1 month after operation (days)	10.5 ± 2.1 (range 6–18)	
Duration of menstruation at 3 months after operation (days)	7.5 ± 1.8 (range 5–12)	
Duration of menstruation at 6 months after operation (days)	7.6 ± 1.6 (range 5–13)	

## Abbreviations

3D: Three-dimensional
LUS: Lower uterine segment.
